# Acoustic Communication in *Dendroctonus adjunctus* Blandford (Curculionidae Scolytinae): Description of Calls and Sound Production Mechanism

**DOI:** 10.3390/insects15070542

**Published:** 2024-07-18

**Authors:** León L. Cerrillo-Mancilla, Claudia Cano-Ramírez, Gerardo Zúñiga

**Affiliations:** Laboratorio de Variación Biológica y Evolución, Departamento de Zoología, Escuela Nacional de Ciencias Biológicas, Instituto Politécnico Nacional, Prolongación de Carpio y Plan de Ayala s/n. Col. Sto. Tomás, Ciudad de México 11340, México; sn_evolve@hotmail.com

**Keywords:** bioacoustics, bark beetle, elytro–tergal, single and multiple–noted calls, withdrawal call

## Abstract

**Simple Summary:**

Simple Summary: Acoustic communication is present in different groups of insects and is used in different contexts during their life cycle. Bark beetles are a group of insects of ecological importance, because they can kill a large number of trees and affect forest succession These insects communicate using chemical signals for long distances, but while on or in the tree, they use acoustic communication to interact between individuals during the colonization of their host trees. A series of sounds have been described for bark beetles, which are produced by specialized anatomical structures under different conditions, such as stress, courtship, and territoriality. In this study, we worked with males of *Dendroctonus adjunctus*, and we described the stridulatory structures using optical and electron microscopy. In addition, we recorded and characterized the types of calls and their spectral and temporal characteristics under stress conditions, female–male, and male–male interactions. Results showed that the shape of the stridulatory apparatus is like males of other beetle species in the genus, and we identified single– and multi–noted calls with differences in temporal and spectral characteristics under three behavioral contexts. Finally, a new type of withdrawal call was identified in male–male interactions.

**Abstract:**

The acoustic communication system (ACS) in bark beetles has been studied mainly in species of the genera *Dendroctonus*, *Ips* and *Polygraphus*. Specifically, ACS of the roundheaded pine beetle, *Dendroctonus adjunctus*, has been little studied. In this study, we described the stridulatory apparatus of this beetle using optical and scanning electron microscopy and recorded the call types produced by males in three behavioral contexts: stress, female–male–, and male–male interactions. From the spectrograms and waveforms, call types, as well as temporal (tooth strike, tooth strike rate, and intertooth strike interval) and spectral features (minimum, maximum and dominant frequency), were determined. Males have a functional elytro–tergal stridulatory apparatus—females do not—consisting of a file for the pars stridens and two lobes for the plectrum. Most of spectro–temporal features were statistically different between single– and multi–noted calls and across the three behavioral contexts. In the male–male interaction, a new type of call named “withdrawal” was produced by the male withdrawing or fleeing. Our results suggest that the spectro–temporal features of single– and multiple–noted calls in the three behavioral conditions are specific and different from each other. Yet, the combination of single and multiple calls determines an overall calling pattern characteristic of the tested behaviors and, therefore, is species–specific.

## 1. Introduction

The acoustic system (AS) (signals, auditory organs, and stridulatory devices) is a key innovation that evolved independently in many insect groups [1,2]. Around 92% of over 195,000 described insect species produce mainly vibrational signals and other forms of mechanical signaling involved in different intra– and interspecific functions [3], such as disturbance and alarm, aggregation, aggression, courtship, copulatory, postcopulatory, and aggressive mimicry [4,5,6,7,8]. Yet, the AS does not consist of a set of evolutionarily independent components, as they are likely to be functionally related to components of other communication systems, such as chemical and visual [9,10,11]. The importance of each system depends on the insect group and on the environment in which they live [1,12].

The pattern of sound signals in insects is varied, complex, and generally species–specific [13]. Sound signals are continuous or discontinuous vibrations with physical properties dependent on its dispersion across a medium and its reception by auditory organs [14,15]. The most common signaling device in insects is an intersegmental stridulatory apparatus, followed by mesonotal–pronotal and other less common apparatuses, such as elytro–tergal, vertex–pronotal, and gula–prosternal [16,17].

Bark beetles (Curculionidae: Scolytinae) are a diverse group of herbivorous insects that play an important role in the ecosystem because they contribute to the structure, composition, dynamics, and vitality of the forests by colonizing and killing old, damaged, or physiologically weakened trees [18,19]. Moreover, bark beetles create food webs by generating habitats by killing trees, which promotes biodiversity (fungi, mites, bacteria, and many other invertebrates and vertebrates use this new resource) [20]. Furthermore, these beetles have established complex ecological interactions with their symbionts, thereby constituting a true holobiont [21,22]. Yet, they are also considered important disturbance agents, because the outbreaks of some bark beetle species can kill thousands of healthy trees and cause a negative impact on ecosystem services for humans and significant economic losses to timber producers [23,24].

Bark beetles are essentially olfactory insects, since they use specific mixtures of semiochemicals to differentiate, select, and colonize host trees and attract mates and conspecifics [25,26]. Inside plants, bark beetles live in a subcortical environment where they build their galleries, mate, and grow their offspring [24]. This is a dark and chemically saturated environment where a chemical communication system could not be reliable [5,15,27]. In the subcortical environment, the AS may be of paramount importance, since wood is a good mediator of sonic signal transmission for communication [27].

Studies of the AS in bark beetles have been carried out mainly in species of the genera *Dendroctonus*, *Ips*, *Hylurgus* and *Polygraphus* [28,29,30,31,32]. From these studies, the manner in which sound is produced in these beetles has been delineated (e.g., elytro–tergal, vertex–pronotal, and gula–prosternal); specifically in the *Dendroctonus*–bark beetles, the mechanism found is the elytro–tergal, which is composed of two structures: the pars stridens located on the inner face of the declive elytral and the plectrum, situated at the seventh abdominal tergite, which generates sound sequences or stridulations [16,28,33]. Moreover, a general catalogue of sounds under stress conditions has been outlined [16], as well as the spectral and temporal characteristics of sound in different biological contexts (e.g., stress, courtship, and female–male interactions) and the effect that some morphological features (e.g., beetle size) have on the sound type produced during courtship [29,30,34,35,36].

The roundheaded pine beetle, *D*. *adjunctus* Blandfort, is an aggressive species that colonize around 18 pine species, preferentially *Pinus hartwegii*, across its distribution range from the United States to Central America [37,38]. Studies of acoustic communication in this bark beetle are scarce and were conducted under a stress condition; in addition, dimorphism was observed in the sound production because only males produced it [16]. Based on this information, we investigated the variability and specific degree of the spectral and temporal features of male calls. We first described the morphology of the stridulatory apparatus using optical and scanning electron microscopy and, later, we recorded the call types produced by males, as well as temporal and spectral features of sounds under different behavioral conditions, such as stress, female–male, and male–male interactions.

## 2. Materials and Methods

*Pinus hartwegii* trees infested and non–infested by the roundheaded pine beetle were felled in May 2023 at the Parque Nacional Volcán Nevado de Colima, Jalisco State, Mexico (19°35′15.2′′ N, 103°36′7.33′′ W; 3414 m elevation). Trees of each condition were sectioned into logs (20 cm length × 30 cm diameter) and sealed at the ends with paraffin to avoid dehydration. Once in the laboratory, uninfected logs were stored in the freezer at 4 °C, while infested logs were enclosed in cloth bags and stored at room temperature. Emerged adults were collected daily, placed in Magenta^TM^ vessels GA 7 (Magenta Corp, Sigma–Aldrich–Merck, Darmstadt, Germany) containing wet filter paper, and stored at 4 °C to keep them alive until needed. The sex of the organisms was ascertained by the shape of the seventh abdominal tergite [39].

### 2.1. Optical and Scanning Electron Microscopy of Stridulatory Structures

**Figure 1:**
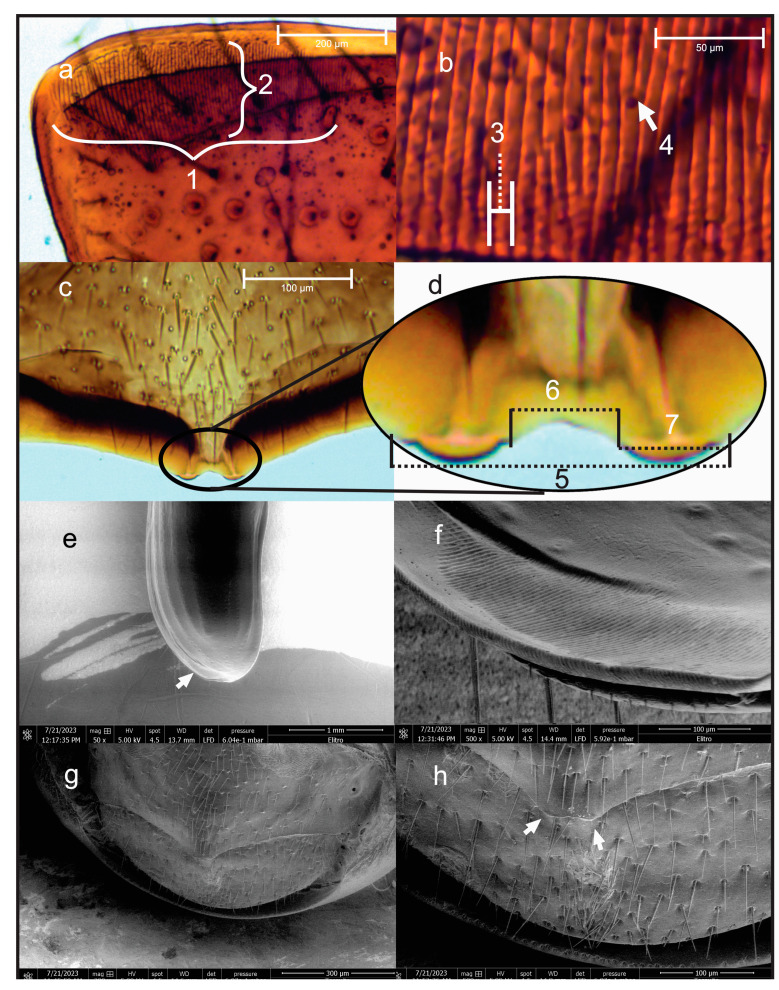
Micrographs of stridulatory apparatus of male *D*. *adjunctus*. (**a**) Pars stridens observed by optical microscopy, (**b**) zoom of pars stridens, (**c**) plectrum observed by optical microscopy, (**d**) zoom of plectrum, (**e**) left elytra and (**f**) pars stridens observed by scanning electron microscopy, (**g**) plectrum observed by scanning electron microscopy, (**h**) zoom of plectrum. The white arrows indicate the pars stridens and the plectrum. (**1**) Length of pars stridens, (**2**) width of pars stridens, (**3**) ridge width, (**4**) division of a ridge, (**5**) external width, (**6**) internal width, and (**7**) lobe width of the plectrum.

**Figure 2:**
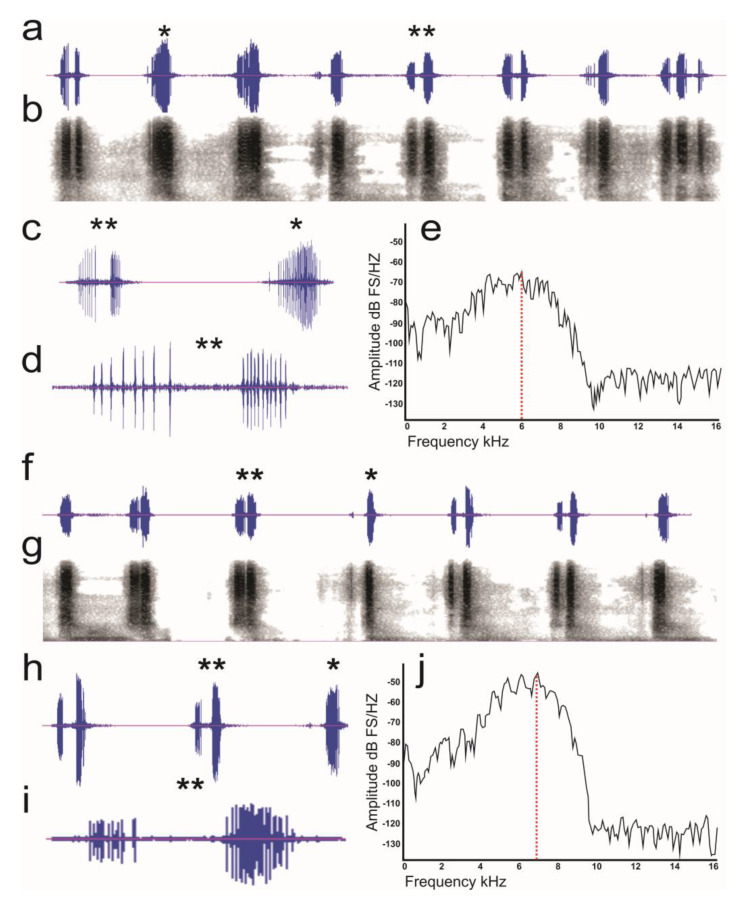
Wave diagram and spectrogram of the stress (**a**–**e**) and female–male (**f**–**j**) condition. (**a**) Wave diagram and (**b**) spectrogram of the stress condition, (**c**) amplification of a single–noted call (*) and an interrupted–noted call (**), (**d**) amplification of an interrupted–noted call, (**e**) relative power diagram, (**f**) wave diagram and (**g**) spectrogram of the female–male interaction, (**h**) amplification of a single–noted call (*) and an interrupted–noted call (**), (**i**) amplification of an interrupted–noted call, (**j**) relative power diagram. The red dashed line indicates the dominant frequency.

**Figure 3:**
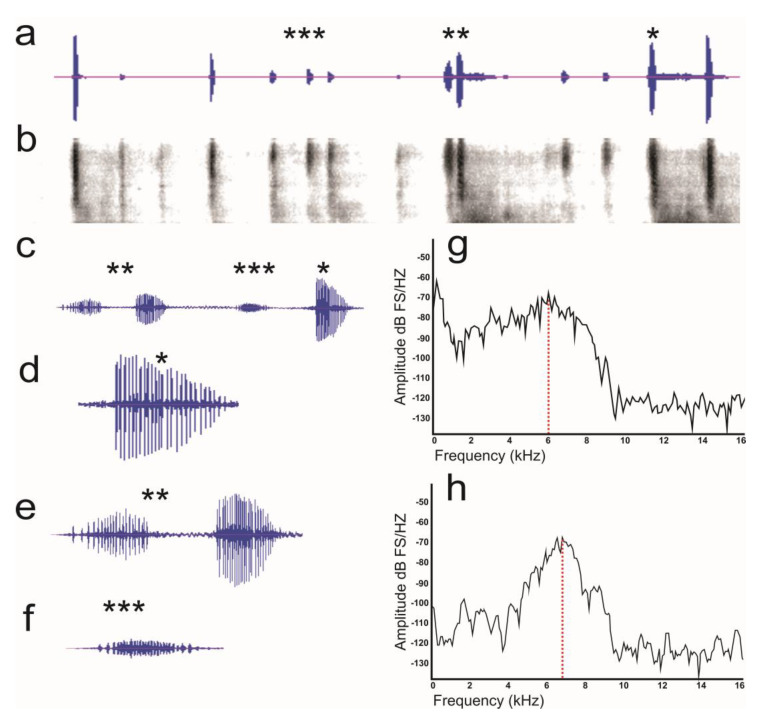
Wave diagram and spectrogram of the male–male condition. (**a**) Wave diagram and (**b**) spectrogram, (**c**) amplification of a single–noted call (*), multiple–noted call (**), and “withdrawal call” (***), (**d**) amplification of an single–noted call, (**e**) amplification of an multiple–noted call, (**f**) amplification of “withdrawal call”(***), (**g**) single–noted call relative power diagram, and (**h**) single–noted call relative power diagram of a “withdrawal call”. The red dashed line indicates the dominant frequency.

The seventh abdominal tergite and the left elytra from 30 males were removed using a pair of fine forceps and fine needles. Both structures were cleared by incubating them for 3 h at 70 °C in a 10% KOH solution. Thereafter, structures were immersed in a 10% acetic acid solution to neutralize the KOH solution, rinsed with 96% alcohol, and later mounted on slides in Hoyer’s medium [40]. The pars stridens (Figure 1a) on the left elytra and the plectrum (Figure 1c) in the seventh tergite were observed by optical microscopy (Prime Star 1, Carl Zeiss, Jena, Germany), and the length and width of both structures were measured with a graduated eyepiece and a 0.01 mm calibration slide (Walfront, Micrometer Calibration Slide). The variables measured of these structures were as follows: The length of the pars stridens was the distance between the ridge closest to the anterior edge of the elytra to the ridge farthest from the posterior edge (Figure 1a-1); the width was the distance from the midpoint of the pars stridens closest to the sutural margin to the farthest midpoint of this structure (Figure 1a-2); and the ridge width was the distance between grooves, calculated from the beginning of one groove to the beginning of the next (Figure 1b-3). The external width of the plectrum was the distance between the outer edge of the right lobe to the outer edge of the left lobe in the anteroposterior position of the insect body (Figure 1d-5). The internal width was the distance between the inner edge of the right lobe to the inner edge of the left lobe (Figure 1d-6), and the lobe width was the distance between the edges of the right lobe (Figure 1d-7). In addition, the stridulatory apparatus of males was observed and photographed in a variable pressure scanning electron microscope under low vacuum (0.6 mbar) and acceleration voltage of 5 kV (FEI Quanta 250 ESEM, FEI Company, Hillsboro, OR, USA) at the Centro de Nanociencias y Micro y Nanotecnologías, Instituto Politécnico Nacional.

### 2.2. Sound Recording

In this study, we referred to the sounds produced by insects as calls [16], which present different temporal and spectral features depending on the type of interaction. To avoid noise disturbance, all calls produced by the roundheaded pine beetle under stress, female–male, and male–male interactions were recorded at night, inside a purpose–built soundproofed wooden box (width × length × depth, 20 × 40 × 30 cm) to minimize stray noise. Calls were recorded with an omnidirectional condenser microphone model ECM8000 (Behringer, Willich, German) and an audio interface model U–PHORIA UM2B (Behringer, Willich, German) in an ASUS laptop, using the virtual audio editor Audacity at 96 kHz, 48 dB gain, and 24 bit sampling rate.

Stress calls of thirty males were recorded while holding them with the thumb and forefinger for 2 min and placing the microphone at a 5 mm distance, leaving the elytra free, and lightly pressing their head and pronotum. Female–male interaction calls were repeated 24 times. For this assay, a 0.9 mm diameter hole was drilled in the non–infested *P*. *hartwegii* logs bark with a drill and a 5/16*4′′ drill bit to go through the bark and avoid reaching the phloem. Subsequently, each female was confined in logs for 1 h by placing an empty gel capsule over the hole. Once the female began to build a gallery, which was confirmed by the accumulation of frass in the entrance hole, the gel capsule was removed and a randomly selected unmated male was placed at the entrance of the gallery [29]. Male–male interaction was repeated 15 times. For this assay, a circular arena of 2 cm diameter was made in the bark of non–infested logs with the help of a knife and a drill with a 5/16 drill bit. Subsequently, the first male was placed in the arena with the help of fine–tipped tweezers. After 1 min, the second male was quickly placed in the arena, covering it with a 0.2 mm mesh to avoid escape. Calls from female–male and male–male interactions were recorded for two minutes or until no signals were detected; the microphone was placed at 2 cm from the hole and arena, respectively [29].

### 2.3. Analyses

#### Spectro–Temporal Features

From the sounds file, call types (single or multiple notes), ratio, and spectro–temporal features were measured using spectrograms and waveforms in Raven Pro™ 1.4 (www.birds.cornell.edu/raven, accessed on 5 March 2023) following the nomenclature from [16,29,40].

A sound call is a train or set of stridulatory impulses provoked by the tooth strike, that is, the rubbing of the plectrum against a tooth of the pars stridens. In bark beetles, single–note calls are characterized by a series of regularly spaced strikes and multiple–noted calls by two or more pulses spaced by brief periods of silence [41]. In this study, multiple–noted calls were considered as sound spacings with a duration longer than two and a half milliseconds, and at least three strikes in each part of the train. The call ratio was calculated as the relationship between single–noted calls or multiple–noted calls with respect to the total number of calls observed; this feature was expressed as a percentage. The temporal features recorded included the tooth strike rate (estimated as the strike number delivered per second), the intertooth strike interval (calculated as the silence time at milliseconds between each tooth strike), and the call duration (estimated as the time that elapsed between the first and last tooth strike from a call). The spectral features evaluated include the maximum frequency (measured as the highest number of repeated waves with respect to time), the minimum frequency (measured as the lowest number of repeated waves), and the dominant frequency (calculated as the overlapping of the frequency with the highest amplitude) [30].

The spectro–temporal features of the single–noted and multiple–noted calls of the three conditions assayed were compared using a paired t–test for independent data and different sample sizes. To estimate significant differences of the spectro–temporal features of single–noted and multiple–noted calls among the three conditions assayed (stress, male–male interaction, and female–male), a one–way ANOVA and post hoc test of Tukey–Kramer were conducted. All analyses were performed with Past 4.03.

## 3. Results

### 3.1. Stridulatory Apparatus of D. adjunctus

The shape of the pars stridens is a file of ridges arranged longitudinally with respect to the inner margin of the elytra. The file has ridges aligned perpendicular to the margin of the elytra, which are highly developed on the left elytra and marginally on the right; in fact, when the right elytra is closed, it overlaps the left (Figure 1a,b,e,f). The pars stridens is characterized by most of its ridges being continuous, with only a minority of them observed as fused at some points (Figure 1b-4). The size of this structure was variable among individuals, with a length of 550–690 ± 7.1 µm, width of 170–260 ± 4.2 µm, and a crest width of 6.25–8.3 ± 0.1 µm (Table 1).

The plectrum is in the middle part of the posterior margin of the seventh tergite; it consists of two conspicuous lobes projecting towards the eight tergite that, when rubbed voluntarily by friction against the pars stridens, produces the sound (Figure 1c,d,g,h). The plectrum was also variable, with an external width of 57–71 ± 0.7 μm, an internal width of 26–31 ± 0.6 µm, and a lobular width of 15–21 ± 0.4 μm (Table 1).

### 3.2. Sound Recording

#### 3.2.1. Stress Calls

Stressed males emitted a call train at an average rate of 2.25 ± 0.07 calls/s and a broadband output of 10–60 dB until the insect was released. The call type was predominantly single–noted in most of the insects (30 specimens) analyzed; however, some of them (13 specimens) also emitted multiple–noted calls (Figure 2a–d; Table 2). The total call ratio was 90% single–noted and 10% multiple–noted (Table 2). The tooth strike number and tooth strike rate per second were higher in single–noted calls than multiple–noted calls; the intertooth strike interval was greater for multiple–noted calls than for single–noted calls. The duration of single–noted calls was slightly shorter than multiple–noted calls (Table 2). Meanwhile, the minimum frequency of single–noted calls was approximately half that of multiple–noted calls, and the maximum and dominant frequencies were similar between both noted calls (Table 2). The plot of the power spectrum showed a single–noted call with a maximum amplitude (dB) between 1 and 9 kHz (Figure 2e).

#### 3.2.2. Female–Male Interaction

In the female–male interaction, twenty four beetles produced single–noted calls, but twelve of them also produced multiple–noted calls in this context (Table 2) (Figure 2f–i). The call rate was 74% single–noted and 26% multiple–noted (Table 2). During courtship, the male initially emitted single–noted calls, but as it approached the female, it produced multi–noted calls. The call train emitted by males was at an average rate of 7.8 ± 0.27 calls/s and an output of 10–65 dB. The number of tooth strikes, the tooth strike per second, and the intertooth strike interval were higher in multiple–noted calls than single–noted calls. The duration of both types of calls was double in multiple–noted calls compared to single–noted calls (Table 2); the minimum, the maximum, and dominant frequencies of spectral parameters were similar between single–noted and multiple–noted calls (Table 2). The plot of the power spectrum showed a single–noted call with an amplitude (dB) between 2 and 10 kHz (Figure 2j).

#### 3.2.3. Male–Male Interaction

Males displayed two behaviors during the interaction: Both males stayed in the arena and later one of them left; the latter behavior was observed in most insects (ten out of fifteen), and the time in which the males retreated from the arena was between 30 to 60 s. The males that remained in the arena emitted a call train at an average rate of 3.7 ± 0.27 calls/s, while males withdrawing emitted a call train at an average rate of 2.56 ± 0.22 calls/s. Calls in both behaviors showed a range of amplitude of 10–60 dB. Single–noted calls were the primary call type in male–male interactions (Table 2) (Figure 3a–d). Males that remained in the arena also produced multiple–noted calls (14.3% of the time; Table 2, Figure 3e). For males that remained in the arena, their temporal and spectral characteristics were maintained during the interaction (Table 2). These temporal and spectral characteristics were like those of the males that remained in the arena until one withdrew from the arena. However, during the insect retraction, the amplitude of the single–noted call decreased significantly.

Significant differences were found between several spectral and temporal features of single–noted and multiple–noted calls generated in each assayed condition. The features tooth strike (*t* = 3.32, *p* = 0.001), tooth strike rate (*t* = 4.68, *p* < 0.05), inter–tooth strike interval (*t* = 5.42, *p* < 0.05), and minimum frequency (*t* = 3.75, *p* < 0.05) were different in the stress condition; tooth strikes (*t* = 3.68, *p* = 0.001), tooth hit rate (*t* = 11.44, *p* < 0.05), intertooth hit interval (*t* = 17. 63, *p* < 0.05), call duration (*t* = 15.54, *p* = 0.04), minimum frequency (*t* = 8.41, *p* < 0.05), and maximum frequency (*t* = 4.83, *p* < 0.05) in the female–male interaction; and only call duration (*t* = 2.11, *p* = 0.04) and dominant frequency (*t* = 2.65, *p* < 0.05) in the male–male interaction (Table 2). ANOVA and the Tukey–Kramer test showed significant differences in the temporal features of the tooth strikes (*F* = 153.0, *p* = 0.001), the frequency of the tooth strikes (*F* = 76.4, *p* = 0.001), the interval between tooth strikes (*F* = 78.58, *p* = 0.001), and the call duration (*F* = 12.29, *p* = 0.001), as well as in the spectral features of minimum (*F* = 68.26, *p* = 0.001) and maximum (*F* = 37.59, *p* = 0.001) frequency both of single and multiple notes among stress, female–male, and male–male conditions (Table 2).

## 4. Discussion

This is the first report of the acoustic diversity of male *D*. *adjunctus* under stress conditions, and female–male and male–male interactions. The roundheaded pine beetle has a stridulatory elytro–tergal apparatus whose general morphological organization aligns with the description of males from *D*. *frontalis*, *D*. *pseudotsugae*, *D*. *brevicomis* [42], *D*. *ponderosae* [29,42], *D*. *rufipennis*, *D*. *valens* [36,42], *D*. *terebrans* [43], *D*. *approximatus* [30], as well as *D*. *rhizophagus*, *D*. *mexicanus*, *D*. *mesoamericanus*, and *D*. *vitei* [44]. No sound was recorded in females of the roundheaded pine beetle under stress conditions, which agrees with the report by [16] for this same species, but not in the female–male interaction. Previous studies have demonstrated that other female *Dendroctonus* have a second stridulatory apparatus called the “terminal abdominal sternite” located in the wall of posterior margin of the last sternite [33,43], from which, it was assumed, they can generate sounds.

To confirm the presence or not of the terminal abdominal sternite, we analyzed 30 females of the roundheaded pine beetle. Interestingly, females have this structure, which is in agreement with that reported for other species of *Dendroctonus* [43,44,45,46], but they also have a structure analogous to the male stridulatory apparatus on the left elytra consisting of a file whose ridges are apparently not well developed and arranged as in males (Figure S1). This structure is also present in females of other *Dendroctonus* species, but it is not known whether they produce sounds with this structure as a true stridulation mechanism [45] as has been reported for *D. terebrans* [43]. It remains to be resolved whether the sounds produced by females are true calls or are acoustic reminiscences of an atrophied morphological structure in the course of evolution, which could be associated with the ecological role that females play as the sex that initiates host tree selection and colonization, as previously suggested [16].

Acoustic sound generation by male *D. adjunctus* includes single– and multi–noted calls in stress tests and female–male and male–male interactions, which are consistent with studies of other *Dendroctonus* bark beetles under the same conditions [16,29,30,36,42,46]. In both call types of this bark beetle, the significant differences observed in spectral and temporal features indicate that these sounds are linked to specific behaviors, as suggested in other *Dendroctonus* species [29,30,36].

Calls produced in stress conditions by the roundheaded pine beetle were mainly 90% single–noted and 10% multiple–noted; these serve as evidence of two different morphotypes: two–noted and three–noted calls, which is consistent with that reported for other *Dendroctonus* bark beetles [16,29,46], including *D. approximatus,* which apparently only generated single–noted calls in stress [30], but our spectrograms indicate that they can also produce multiple–noted distress calls (Figure S2). In addition, our findings also showed that the intraspecific variation of spectro–temporal features in the roundheaded pine beetle was low and independent of note type and multi–note morphotypes. However, significant differences were found between spectro–temporal features in both call types (Table 2), except maximum and dominant frequencies, and call duration. Furthermore, the interspecific comparison of some spectral–temporal features (e.g., maximum, minimum, and dominant frequencies) of different species generated in stress (Table 3) suggest that the patterns of single–noted distress calls are similar among them, except for the call duration records reported for *D*. *approximatus* and *D*. *terebrans* [16]. Unfortunately, the absence of multi–noted call data does not allow us to evaluate whether the spectro–temporal features of these call types are similar or different between species.

Significant differences between the spectro–temporal features of single–noted and multiple–noted calls produced by *D*. *adjunctus* males in the female–male interaction and in the stress condition indicate that the general pattern of these calls corresponds with different biological behaviors. Our results show a higher prevalence of single notes than multiple notes in this female–male interaction, which does not coincide with that observed in *D*. *ponderosae* and *D*. *approximatus* where multiple–note calls are predominant [29,30]. Unfortunately, it is also not possible to compare spectral and temporal features between species, as these data have not always been reported.

It has been suggested that the production of interrupted calls is indicative of the vigor and fitness of males, which determines their preference for females [36]. Nevertheless, the ratio of both types of calls, the emission pattern, and their temporal combination or alternation may also be a mechanism of intraspecific recognition and of interspecific reproductive isolation, especially when several species coexist in sympatry or syntropy. This is because it is widely recognized that the *Dendroctonus* species can coexist in space and time in the same locality and tree. Some studies have shown that males approaching the female’s gallery produce calls that cause the female to stop producing aggregation pheromones [45]. This may be associated with the colonization and mass attack strategy of these species.

Male–male interaction is a special case of stress to avoid physical aggression, which can impede the access of another male to the female or to block his entry into the gallery (territoriality). Although the proportion of single and multiple calls between the stress condition (90–10%) and the male–male interaction (85–15%) was similar in this study, significant differences between both conditions were mainly concentrated in temporal characteristics (e.g., rate of tooth strikes, interval between tooth strikes, and call duration), suggesting the configuration of different calling patterns in these two stress conditions. The pattern of calls in the male–male interaction could be due to combinations of intimidation or deterrence to avoid physical contact. In fact, the proportion of single– and multi–note calls was similar in males that remained in the arena but not in males that withdrew, which were only single–note. Similar results have been obtained in *D. ponderosae* and were interpreted as a characteristic of rivalry between individuals of the same sex, regardless of the presence of a female or of maintaining or blocking the entrance to the gallery when occupied by a pair [29]. In contrast, in *D*. *valens*, the sound pattern produced by males was related to their body size, as males competing for females displayed two types of calls, the first was to prevent direct fighting with potential competitors of equivalent size, while the second was to scare away and deter small–sized competitors [47].

In the male–male interaction, both males of *D*. *adjunctus* produced both types of calls while staying together, but when one of the males withdrew, not only did its aggressive behavior change, but the call sound also changed to being 100% one–noted. The minimum frequency of retreat calls was twice that of calls made by males while interacting in territoriality, the maximum frequency was lower in retreat calls compared to the calls of males that stayed together, and the dominant frequency was very similar across all interactions (Table 2). Differences between the calls of males that remained and those that withdrew showed a wider repertoire of signals compared to those known so far, introducing a new type of sound, the “withdrawal calls”. These calls, when exhibited in conjunction with flight behavior, may be an indicator of surrender by the male. Future studies may provide clarity on the behavioral implications of these types of calls.

Lastly, while the AS (signals, auditory organs, and stridulatory devices) in other insect orders (e.g., Orthoptera, Hymenoptera, and Hemiptera) have been widely studied and associated with a wide range of biological behaviors and environmental factors, in bark beetles (Coleoptera: Curculionidae: Scolytinae), its integration with the chemical communication system, behavior, and reproductive ecology has received little attention [1,33,48,49]. In particular, it would be desirable for future studies focus on aspects related to acoustic signals, which, being apparently species–specific, could be involved in isolation and reproductive behavior, as well as in pheromone synthesis, especially in species that produce these compounds de novo.

## Figures and Tables

**Table 1:** Measurements of the stridulatory apparatus, pars stridens, and plectrum in males of *D*. *adjunctus*.

* Pars Stridens	**Width (µm)**	**Length (µm)**	**Ridge Width (µm)**
Range	170–260	550–690	6–8
Media	219.71	610.85	6.71
Standard error	25.26	42.31	0.78
* Plectrum	**Internal width (µm)**	**External width (µm)**	**Lobe width (µm)**
Range	26–31	57–71	15–21
Media	26.54	65.12	19.56
Standard error	3.74	4.73	2.64

* See Figure 1 for information about plectrum and pars stridens features.

**Table 2:** Temporal and spectral measurements of single–noted and multiple–noted calls of *Dendroctonus adjunctus* in three interactions: stress, female–male, and male–male interactions.

Call Context	Call Type	n	Proportion (%)	Temporal					Spectral			
				Tooth Strike (No./Call)	Tooth Strike Rate (No./s)	Intertooth Strike Interval (ms)	Call Duration (ms)	Minimum Frequency (kHz)		Maximum Frequency (kHz)		Dominant Frequency (kHz)
Stress	Single	30	0.9	41.4 ± 0.7	1034.3 ± 26.5	0.9 ± 0.03	40 ± 1	2.1 ± 0.14	a	8.6 ± 0.03	a	6.5 ± 0.06
	Multiple	13	0.1	27.6 ± 6.5	585.7 ± 75.2	1.6 ± 0.23	47.2 ± 5.5	4.3 ± 0.04		8.7 ± 0.08		6.7 ± 0.3
Female–male	Single	24	0.74	23.8 ± 1.8	675 ± 20.7	1.4 ± 0.04	35.3 ± 3.7	5.6 ± 0.07	b	7.8 ± 0.08	b	6.7 ± 0.07
	Multiple	12	0.26	36.9 ± 1.9	532 ± 27.4	1.8 ± 0.09	69.5 ± 1.3	4.3 ± 0.1		7.1 ± 0.07		6 ± 0.06
Male–male remains	Single	15	0.857	16.4 ± 1	611.7 ± 15.5	1.6 ± 0.04	26.8 ± 2	2.7 ± 0.19	a	8.4 ± 0.07	c	6.6 ± 0.06
	Multiple	7	0.143	21.9 ± 0.3	549.6 ± 24	1.8 ± 0.07	40 ± 1.5	2.5 ± 0.41		8.5 ± 0.26		6.2 ± 0.11
Male–male withdrawal	Single	10	1	24 ± 1.9	616.3 ± 24	1.6 ± 0.05	39 ± 2.8	5 ± 0.21	b	7.3 ± 0.11	d	6.3 ± 0.11
	Significance level Paired t–test											
Comparison between call type	Stress		*t=*	3.32 (*p* < 0.05)	4.68 (*p* < 0.05)	5.42 (*p* < 0.05)	1.84 (*p =* 0.06)	3.75 (*p* < 0.05)	1.05 (*p* = 0.2)		1.04 (*p* = 0.3)	
	Female–male		*t=*	3.68 (*p* < 0.05)	11.44 (*p* < 0.05)	17.63 (*p* < 0.05)	15.54 (*p* < 0.05)	8.41 (*p* < 0.05)	4.83 (*p* < 0.05)		5.88 (*p* = 0.3)	
	Male–male remains		*t=*	1.68 (*p* < 0.05)	1.66 (*p* = 0.1)	1.58 (*p* < 0.1)	2.11 (*p* < 0.05)	0.44 (*p* = 0.6)	0.66 (*p* = 0.5)		2.65 (*p* < 0.05)	
	Significance level one way ANOVA											
Comparison between context	Single		*F=*	153 (*p* < 0.05)	76.4 (*p* < 0.05)	78.58 (*p* < 0.05)	12.29 (*p* < 0.05)	68.26 (*p* < 0.05)	37.59 (*p* < 0.05)		2.7 (*p* = 0.06)	
	Multiple		*F=*	3.4 (*p* < 0.05)	17.76 *(p* < 0.05)	42.69 (*p* < 0.05)	238.3 (*p* < 0.05)	19.58 (*p* < 0.05)	42.71 (*p* < 0.05)		4.1 (*p* = 0.08)	

Condition with significant difference for each temporal characteristic and treatments associated with the same lowercase letter did not differ significantly for spectral characteristics, through the Tukey–Kramer test.

**Table 3:** Temporal and spectral measurements of the single–noted and multiple–noted calls in different species of the genus *Dendroctonus*.

Species			Temporal				Spectral			Reference
			Tooth Strike (No./Call)	Tooth Strike Rate (No./s)	Intertooth Strike Interval (ms)	Call Duration (ms)	Minimum Frequency (kHz)	Maximum Frequency (kHz)	Dominant Frequency (kHz)	
*Dendroctonus adjunctus*	Stress	Single–noted	41.4 ± 0.7	1034.3 ± 26.5	0.9 ± 0.03	40 ± 1	2.1 ± 0.14	8.6 ± 0.03	6.5 ± 0.06	Our data
		Multiple–noted	27.6 ± 6.5	585.7 ± 75.2	1.6 ± 0.23	47.2 ± 5.5	4.3 ± 0.04	8.7 ± 0.08	6.7 ± 0.3	
	Female–male	Single–noted	23.8 ± 1.8	675 ± 20.7	1.4 ± 0.04	35.3 ± 3.7	5.6 ± 0.07	7.8 ± 0.08	6.7 ± 0.07	
		Multiple–noted	36.9 ± 1.9	532 ± 27.4	1.8 ± 0.09	69.5 ± 1.3	4.3 ± 0.1	7.1 ± 0.07	6 ± 0.06	
	Male–male	Single–noted	16.4 ± 1	611.7 ± 15.5	1.6 ± 0.04	26.8 ± 2	2.7 ± 0.19	8.4 ± 0.07	6.6 ± 0.06	
		Multiple–noted	21.9 ± 0.3	549.6 ± 24	1.8 ± 0.07	40 ± 1.5	2.5 ± 0.41	8.5 ± 0.26	6.2 ± 0.11	
*Dendroctonus ponderosae*	Stress	Single–noted	17.4 ± 1.8	828.5 ± 59.8	1.4 ± 0.1	21.8 ± 1.7	ND	ND	15.6 ± 2.8	[29]
		Multiple–noted	27.9 ± 4.1	593.2 ± 89.5	2.2 ± 2.8	56.3 ± 8.0	ND	ND	18.3 ± 5.8	
	Female–male	Single–noted	21.3 ± 3.5	786.0 ± 63.7	1.4 ± 0.1	30.0 ± 6.8	ND	ND	26.0 ± 4.6	
		Multiple–noted	35.0 ± 3.5	433.1 ± 18.8	2.6 ± 0.1	90.0 ± 6.5	ND	ND	21.9 ± 5.9	
	Male–male	Single–noted	16.9 ± 2.2	709.5 ± 40.2	2.6 ± 1.0	28.8 ± 6.3	ND	ND	17.4 ± 1.6	
		Multiple–noted	22.2 ± 2.2	464.0 ± 44.7	2.6 ± 0.2	56.1 ± 8.5	ND	ND	26.4 ± 3.4	
*Dendroctonus approximatus*	Stress	Single–noted	55.1 ± 0.6	517.6 ± 23.0	ND	108.6 ± 1.2	ND	ND	5.2 ± 0.1	[30]
	Female–male	Single–noted	61.7 ± 1.2	505.7 ± 12.0	ND	124.5 ± 2.7	ND	ND	5.6 ± 0.12	
*Dendroctonus adjunctus*	Stress	Single–noted	ND	ND	ND	39.0 ± 12.2	2.99 ± 0.13	10.3 ± 1.68	5.94 ± 1.94	[16]
*Dendroctonus brevicomis*	Stress	Single–noted	ND	ND	ND	68.1 ± 27.4	3.86 ± 0.74	14.3 ± 4.21	6.03 ± 1.50	
*Dendroctonus frontalis*	Stress	Single–noted	ND	ND	ND	59.9 ± 19.2	3.85 ± 0.73	18.2 ± 3.84	7.99 ± 4.36	
*Dendroctonus pseudotsugae*	Stress	Single–noted	ND	ND	ND	36.4 ± 8.8	2.86 ± 0.42	8.16 ± 2.03	4.92 ± 1.25	
*Dendroctonus terebrans*	Stress	Single–noted	ND	ND	ND	99.3 ± 21.9	6.08 ± 2.23	39.2 ± 3.57	22.6 ± 6.05	
*Dendroctonus terebrans*	Stress	Single–noted	11.3 ± 0.6	375 ± 79	ND	31 ± 6	ND	ND	ND	[47]

ND = Not determined.

## Data Availability

All relevant data for this study are reported in this article.

## Supplementary Materials

The following supporting information can be downloaded at: https://www.mdpi.com/article/10.3390/insects15070542/s1, Figure S1: Structure of the stridulatory apparatus of *Dendroctonus adjunctus* female. (**a** and **b**) left elytra, (**c**) absence of plectrum, and (**d**) pars stridens in the last sternite; Figure S2: Wave diagram and spectrogram of the male of *Dendroctonus approximatus* in stress condition. (**a**) Wave diagram, single-noted call (*), multiple-noted call (two note ** and three note ***), and (**b**) spectrogram of the stress condition, amplification of a (**c**) single-noted call and multiple-noted call of (**d**) two note and (**e**) three note, and (**f**) single-noted call relative power diagram.
