# Supplementary material for: Acoustic Communication in Dendroctonus adjunctus Blandford (Curculionidae Scolytinae): Description of Calls and Sound Production Mechanism
Source: Insects. 2024 Jul 18;15(7):542. doi: 10.3390/insects15070542 (PMC11277085; doi:10.3390/insects15070542)
Supplement: Supplementary file 1 [file insects-15-00542-s001.zip › insects-3100018-supplementary.pdf]

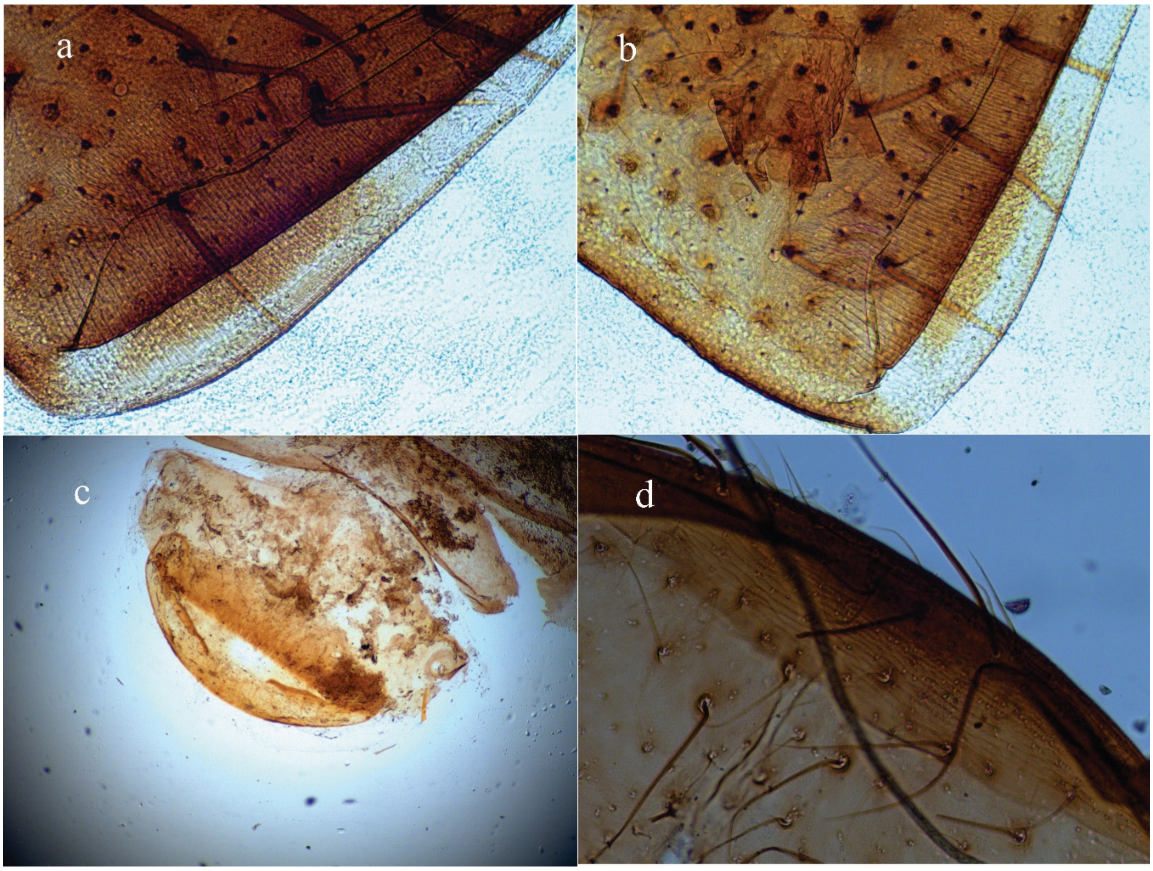

Figure S1. Structure of the stridulatory apparatus of *D*. *adjunctus* female. (**a** and **b**) left elytra, (**c**) absence of plectrum, and (**d**) pars stridens in the last sternite.

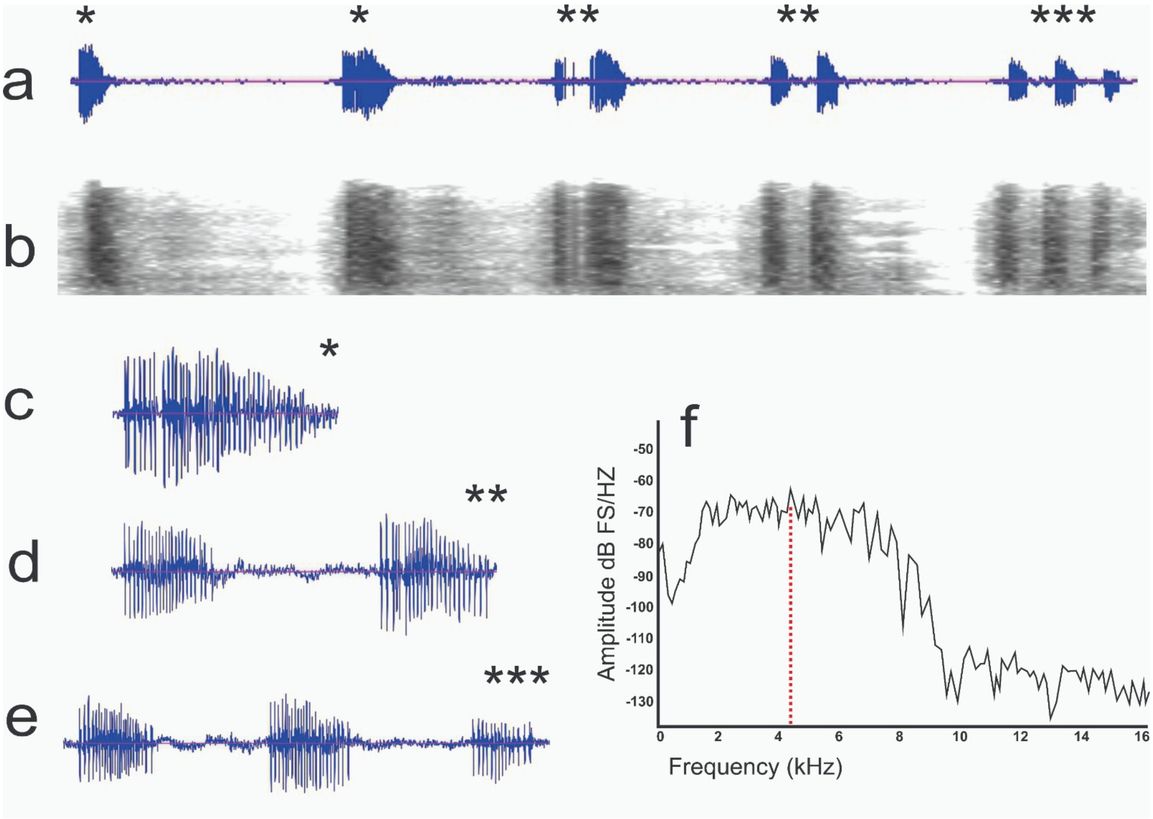

Figure S2. Wave diagram and spectrogram of the male of *Dendroctonus approximatus* in stress condition. (**a**) Wave diagram, single-noted call (*), multiple-noted call (two note ** and three note ***), and (**b**) spectrogram of the stress condition, amplification of a (**c**) single-noted call and multiple-noted call of (**d**) two note and (**e**) three note, and (**f**) single-noted call relative power diagram.
